# Conjunctival granulomatous capillary haemangioma in children: case report and review of the literature

**DOI:** 10.1186/s12887-021-02924-5

**Published:** 2021-10-11

**Authors:** Caiping Shi, Yanhong Ren, Jia Feng, Weizhong Guo, Xiaoyu Zheng

**Affiliations:** The Children’s Hospital of Zhejiang University School of Medicine, National Clinical Research Center for Child Health, 3333 Binsheng Rd, Zhejiang, 310052 Hangzhou China

**Keywords:** Conjunctival tumour, Granulomas, Haemangioma, Capillary haemangioma, Lobular haemangioma

## Abstract

**Background:**

Granulomatous capillary haemangioma refers to a benign vascular tumour that commonly affects the skin, with occasional involvement of the mucosa. Reports of conjunctival granulomatous capillary haemangioma in children are uncommon. In this article, we present a case of granulomatous capillary haemangioma and a brief review of the relevant literature.

**Case presentation:**

An 11-year-old girl presented with a conjunctival mass. An excision of the entire lesion was performed. Histopathology showed a granulomatous capillary haemangioma.

**Conclusions:**

The clinical manifestations of granulomatous capillary haemangioma lack specificity; pathological characteristics and immunohistochemistry are the main basis for diagnosis. We retrospectively analysed the diagnosis and treatment of a patient with conjunctival granulomatous capillary haemangioma to deepen the understanding and facilitate the diagnosis and treatment of this disease.

## Background

Granulomatous capillary haemangioma refers to a benign vascular tumour that commonly affects the skin, with occasional involvement of the mucosa [1]. However, such disease occurring in the eye is relatively rare in children. Various factors are implicated in the etiopathogenesis of this entity, but the actual mechanism of granulomatous capillary haemangioma is not very clear. Different investigators have suggested various aetiologic factors, such as trauma, [2] hormonal factors, [3] bacteria or viruses and certain drugs [3]. We retrospectively analysed the diagnosis and treatment of a patient with a conjunctival granulomatous capillary haemangioma near the caruncle to explore the clinical and pathological characteristics and thereby deepen the understanding and facilitate the diagnosis and treatment of this disease.

## Case presentation

**Fig. 1:**
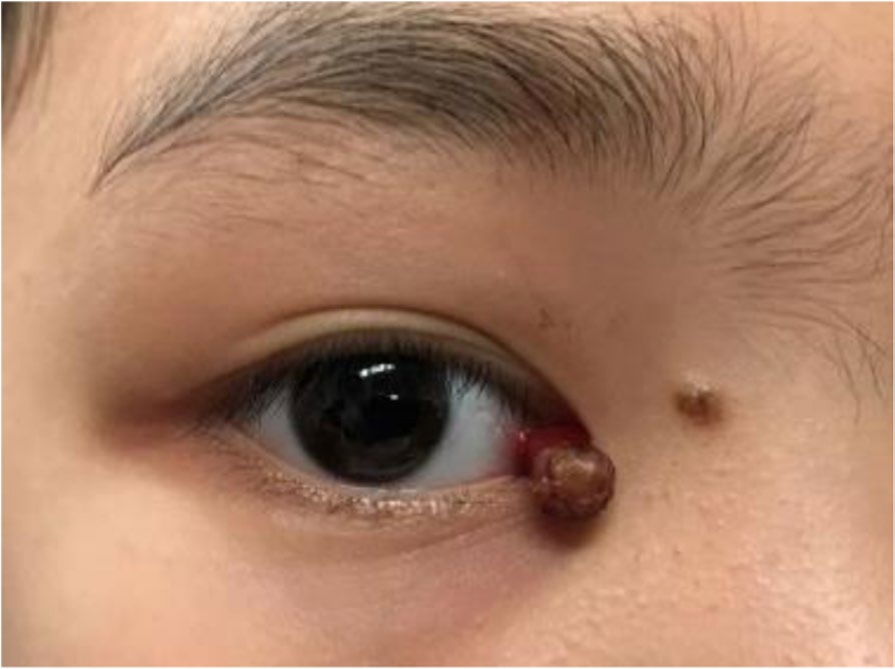
Preoperative mass

**Fig. 2:**
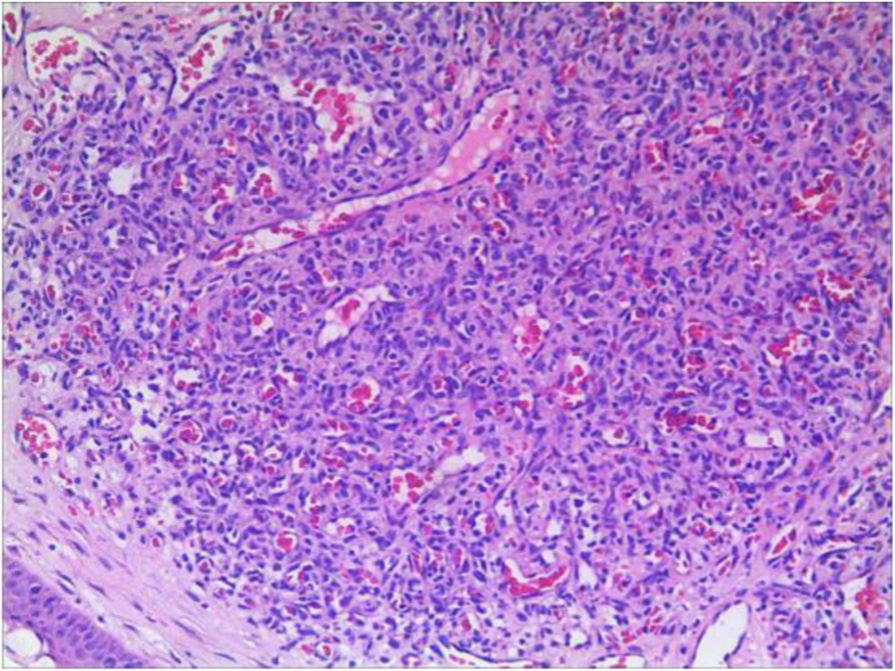
HE50

**Fig. 3:**
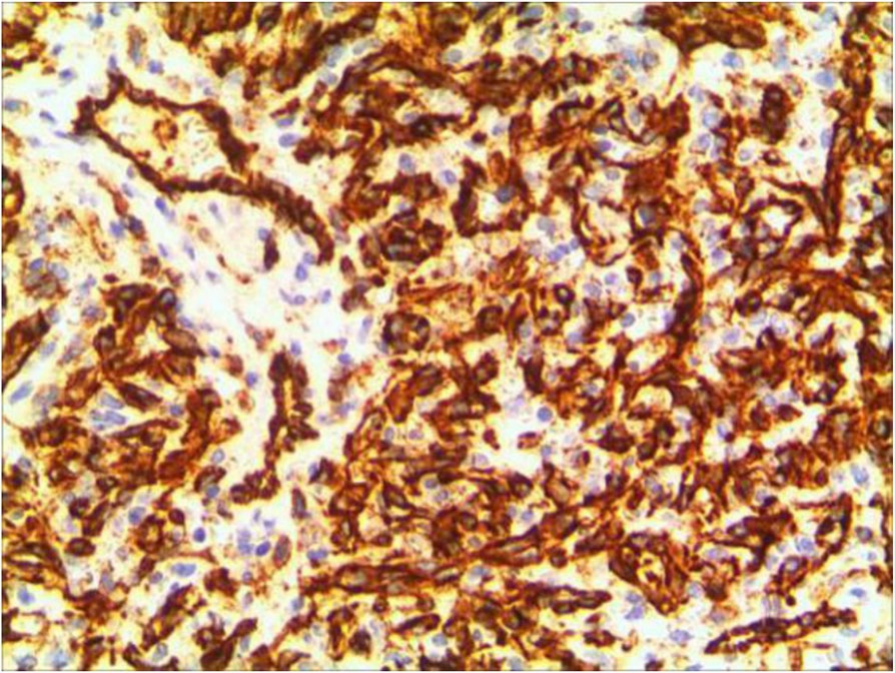
CD31

**Fig. 4:**
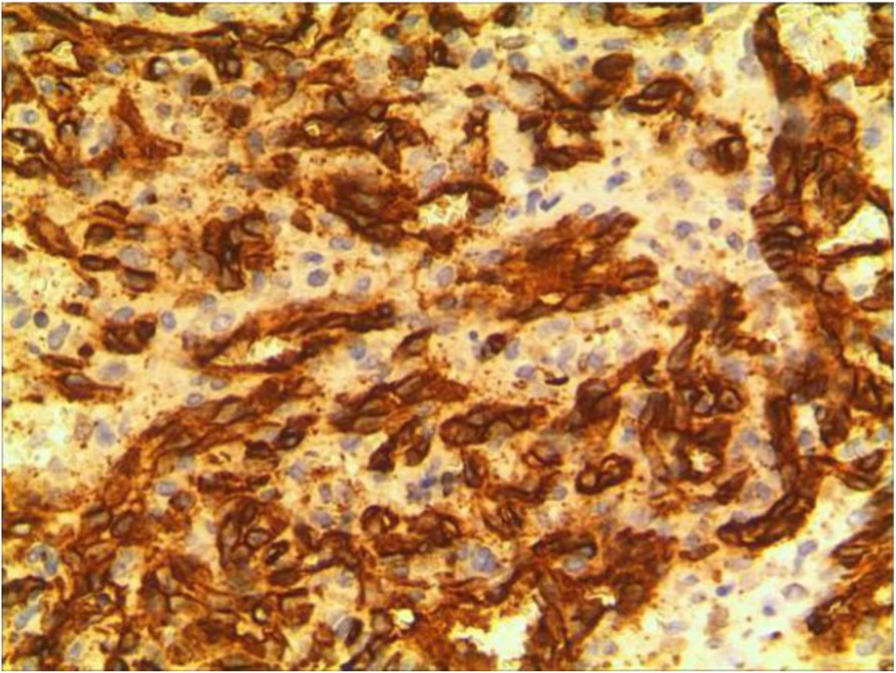
CD34

**Fig. 5:**
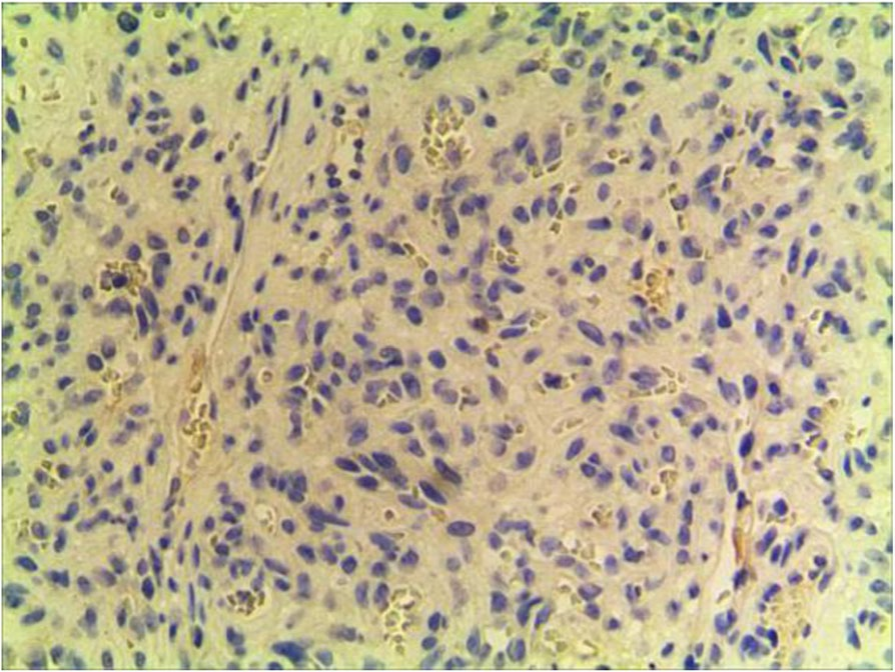
D2–40

**Fig. 6:**
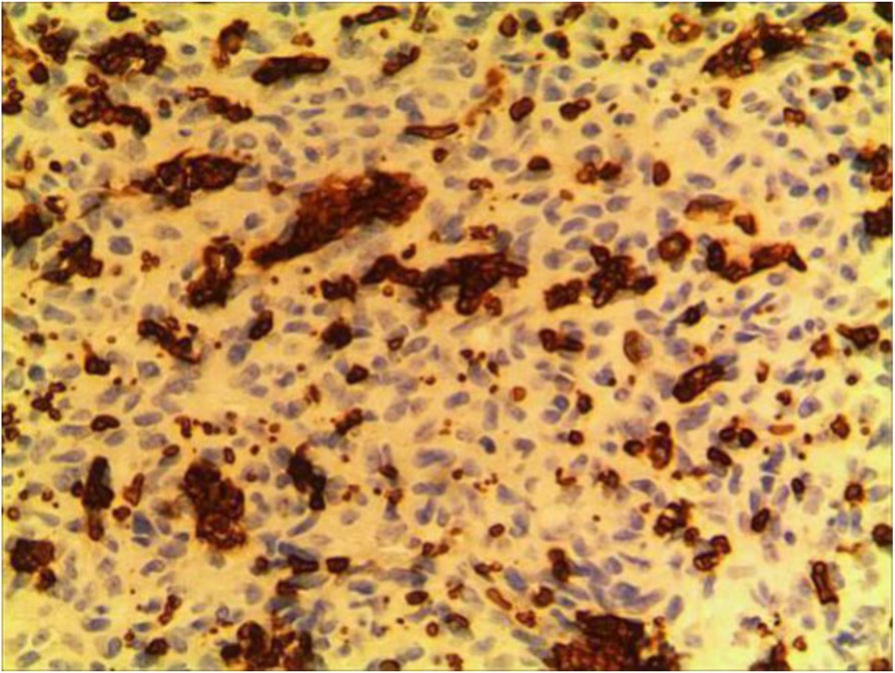
GLUT1

**Fig. 7:**
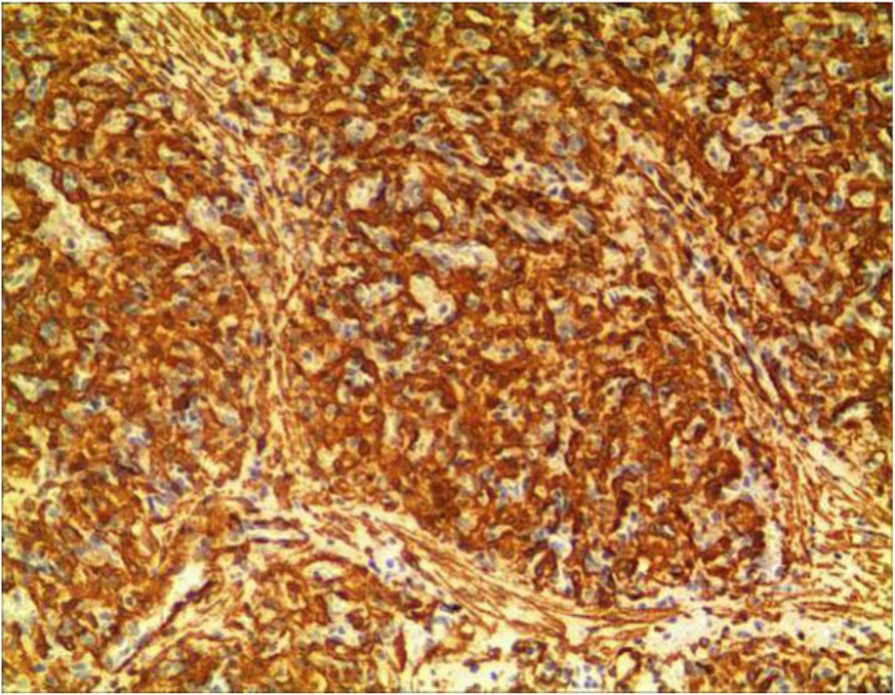
SMA+

**Fig. 8:**
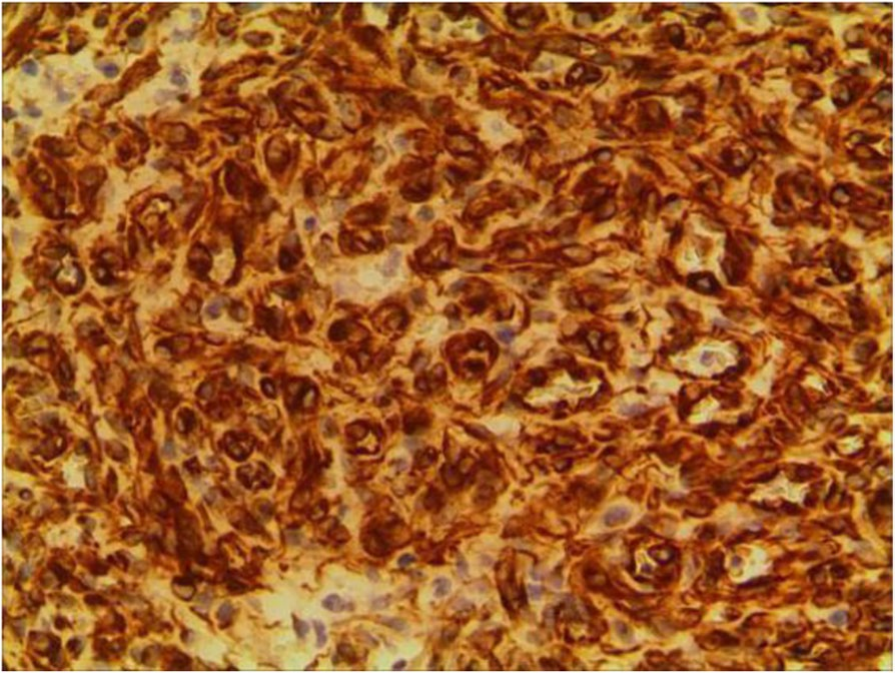
WT1

**Fig. 9:**
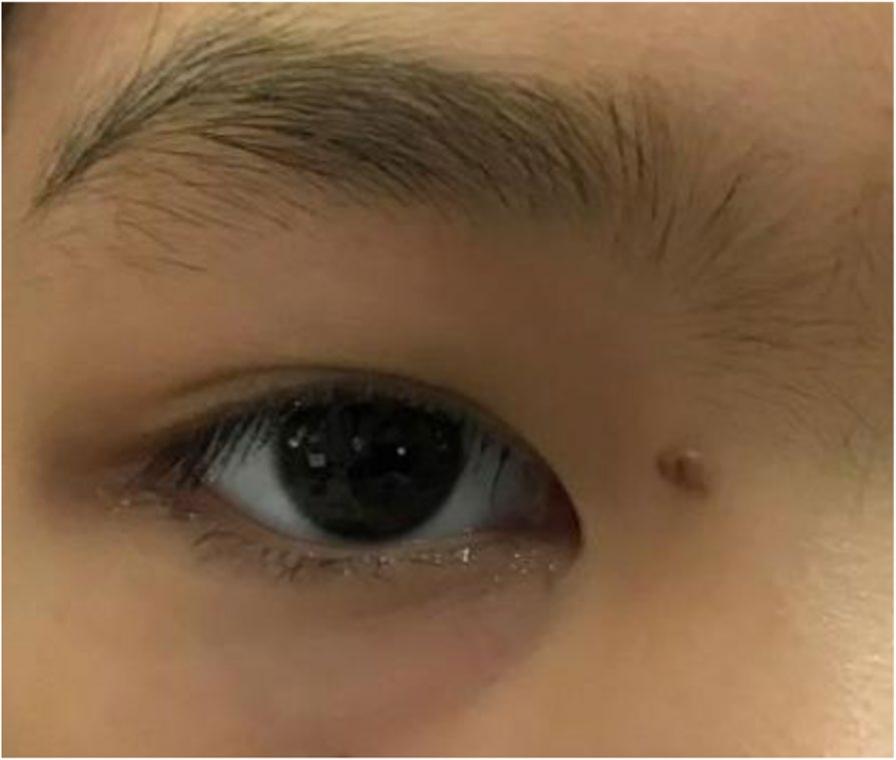
Postoperative appearance

An 11-year-old girl with no prior history of ocular trauma, surgery, or vascular lesions presented with a 1-month history of swelling lesions involving the conjunctiva of the right eye. There was a swelling bulge of approximately 3 mm × 3 mm in the corner of the right eye that had led to increased secretion, itching, and rubbing. After treatment with levofloxacin eye drops, the secretion was reduced, but the swelling and itching continued with slight bleeding. Bleeding occurred intermittently, and the tumour increased gradually. When the patient registered at our hospital, the bulge was increased. Physical examination revealed normal vision in the two eyes and a red mass of approximately 10 mm × 5 mm that protruded from the conjunctiva near the inner canthus without eyelid swelling; and no conjunctival hyperaemia (Fig. 1). The mass was brittle with slight oozing and a pedicle connecting to the conjunctiva, whose surface seemed to have sleeve-like scabs with pus-like adhesion. The boundary of the mass was clear. The anterior chambers and the pupils of both eyes were normal. The conjunctival cylindrical mass was excised completely during general anaesthesia. The peripheral and basal tissues around the mass were brittle and easily bled. When the mass was removed, vascular proliferation around the base could be seen near the caruncle and was destroyed by cauterization. With regular dressing changes daily, the wound healed nicely. The pathology report showed that the surface was covered with squamous epithelium, the interstitium showed clustered proliferation with small blood vessels, and there was a small amount of inflammatory necrotic exudate. Immunohistochemistry results were as follows: Glut(−), WT-1(+), SMA(+), CD 31(+), CD 34(+), D2–40(−). This was consistent with granulomatous capillary haemangioma (Figs. 2, 3, 4, 5, 6, 7, 8). After 6 months of follow-up, there was no recurrence (Fig. 9).

## Discussion and conclusions

Granulomatous capillary haemangioma is a benign vascular proliferative disease involving the skin or, occasionally, the mucous membranes. In 1904, the disease was named pyogenic granuloma (PG) by Hartzell et al .[4] In 1980, Mills called it lobular capillary haemangioma (LCH ) [5]. There are other names include Crocker and Hartzell’s disease, granuloma pyogenicum, granuloma pediculatum benignum, benign vascular tumor and granuloma gravidaru [4]. In fact, this haemangioma is not a true tumour, and it is essentially different from a true haemangioma [6].

Granulomatous capillary haemangioma is a vascular nodule composed by capillary hyperplasia, which is present on the surface of the skin or mucous membrane; and often occurs in exposed parts of the body that are vulnerable to trauma. The commonly involved sites are on the head, face, lips and maxillofacial region, limbs, trunk, nasal cavity, auricle, palate, vocal cords, etc. Granulomatous capillary haemangioma may be seen in patients of all ages. The literature reports on the eye have mainly involved adults, usually after surgery or conjunctival trauma, such as symblepharon release, strabismus surgery or enucleation, and the disease is known to mainly occur in the eyelids, conjunctiva or cornea [7–11] of adults, but seldom in children. The mass shows rapid exophytic growth with surface ulceration. It usually happens singly with a fixed base, which easily bleeds after touching.

Many factors are implicated in the etiopathogenesis of this entity, but the exact cause is unknown. Approximately 1/3 of the cases are related to trauma. Infection is also known to be an related factor, mainly caused by *Staphylococcus aureus* or *Streptococcus*. Hormonal factors may play a role in pregnancy with granulomatous capillary haemangioma. There are some cases of granulomatous capillary haemangioma within the oral cavity reported in the literature to occur during pregnancy, suggesting that the pathogenesis may be related to the synergistic effect of oestrogen and progesterone [12]. In the present case, there was no prior history of ocular trauma, but there were infection-related manifestations such as excessive ocular secretions, frequent eye rubbing, and bleeding, which may be related factors.

The history and physical examination of granulomatous capillary haemangioma are not specific, so such cases are often misdiagnosed. A granulomatous capillary haemangioma typically starts as a small, red papule. Then, it undergoes a variable, sometimes rapid, exophytic growth phase over several weeks to months. The tumour often shows frequent ulceration and bleeding, which is the usual event that warrants treatment. This disease is mainly differentiated from inflammatory granuloma, juvenile haemangioma, papilloma, epithelioid haemangioendothelioma, Kaposi’s sarcoma and other malignant tumours. The means of identification mainly rely on pathological examination and immunohistochemical analysis.

The pathological characteristics of granulomatous capillary haemangioma are as follows: the lesion is mostly exogenous; and located in the mucosa or dermic tissues with a nonenveloped lobular structure; but with clear boundaries. An ulcer is often formed with the infiltration of a large number of acute and chronic inflammatory cells and the formation of abundant granulation tissue, interstitial porosity and oedema; however, the deep lobular structure is still clear. The lesion stains positive for vascular markers such as CD31, CD34, and factor VIII antigen; but negative for glucose transporter-1 (GLUT1 ) [13, 14]. Human endothelial receptor tyrosine kinase Tie2 plays an important role in the development and progression of pyogenic granulomas in the presence of alpha SMA antibodies [15, 16]. A study on the pathology of 16 adult periocular granulomatous capillary haemangiomas showed that all cases had similar histopathological findings: lesions composed of proliferating capillary lumens surrounded by endothelial cells but not inflammatory cells and epithelioid giant cells [11].

Differentiation from inflammatory granuloma: The capillaries of inflammatory granuloma exhibit directional, bud-like growth and no lobular structure; inflammatory granuloma has more obvious inflammation, while granulomatous capillary haemangioma has less inflammation.

Differentiation from capillary haemangioma: Capillary haemangioma mainly occurs in infants or young children with proliferating capillaries and endothelial cells in more than 2 layers that have atypical and mitotic features, and the tumour can involve subcutaneous and submucosal tissues with little interstitium. However, there is no lobular structure.

Differentiation from papilloma: The papilloma is pale or light red with a polypoid or papillary appearance; the pathological feature is high hyperplasia of epithelial tissue and excessive growth of squamous epithelium to form papillae. The centre has loose and vascularized connective tissue.

Differentiation from epithelioid haemangioma: Epithelioid haemangiomas can be nodular or lobular structures, with endothelial cells that are like “shoe spikes” protruding into the vascular cavity as epithelioid cells. There is obvious infiltration of interstitial inflammatory cells mainly consisting of eosinophils and lymphocytes.

Differentiation from Kaposi’s sarcoma: This tumour, which is closely related to HIV, can grow in a nodular fashion with spindle-shaped cells that are moderately atypical and distributed in pieces. There are vascular fissures among the cells.

Differentiation from well-differentiated angiosarcoma: The boundary of this lesion is unclear without a lobular structure. The vascular cavities are irregular and coincide with each other. The cells of the lesion are atypical.

In summary, the pathological feature of granulomatous capillary haemangioma is that it occurs in the mucosa or dermis and has a lobular structure and clear boundaries. It is formed by interstitial oedema; and inflammatory cell infiltration; epidermal cells become thin or ulcerated and form a granuloma with the proliferation of spindle cells around blood vessels. Immunohistochemistry mainly includes vascular endothelial cells and perivascular spindle cells. In most cases, the perivascular spindle cells are more SMA+, and the vascular endothelial cells are more CD34 + .

At present, there are various treatment methods for granulomatous capillary haemangioma. Regardless of the treatment, the patient should be counselled about the risk of recurrence. Complete excision is the best method because of lower rates of recurrence and the obtainment of an excellent specimen for histopathologic characterization. Other nonsurgical avenues include cryotherapy, electrocautery, chemical cautery with silver nitrate without excision, or laser therapy. Patients who still relapse after surgery can be treated by local intralesional injection with triamcinolone acetonide. Drugs mainly include sclerosing agents (urea, pingyangmycin and cinnamyl alcohol) and topical β-blockers. Injecting polidocanol to treat granulomatous capillary haemangioma is more common and useful in the auricle, forehead, lower lip, fingers, face, limbs and trunk [17–20]. Surgery excision is mainly applied in the eye. There are reports in the literature that topical timolol eye drops have been used for treatment with good results and no side effects [21–24]. There are various surgical methods for granulomatous capillary haemangioma, such as direct curettage, excision and suture, and local flap transfer according to the size of the tumour. Although granulomatous capillary haemangioma is a benign tumour, a recurrence rate of approximately 16% has been reported. In the present case, the patient underwent thorough resection and anti-inflammatory treatment after the operation. The wound healed well. There was no sign of recurrence after 6 months of follow-up, and the prognosis was good.

Granulomatous capillary haemangioma is rare, and the pathogenesis is unclear. Moreover, its clinical manifestations are not specific. The diagnosis mainly depends on pathology and immunohistochemistry. This case reports the above characteristics; to promote a clear diagnosis and timely treatment, which yields a good prognosis after active surgical resection.

## Data Availability

All relevant data are included in this manuscript and associated figures. However, if more information is required, the datasets analysed for the current study are available from the corresponding author upon reasonable request.

## Abbreviations

PG: Pyogenic granuloma
LCH: Lobular capillary haemangioma
GLUT1: Glucose transporter-1
